# Broadband NIR-II Emission with Wide Excitation Range in Cs_2_WCl_6_ Double Perovskites Utilizing Re^4+^ Doping

**DOI:** 10.3390/nano16070400

**Published:** 2026-03-26

**Authors:** Yu Xiao, Xiaobo Hu, Ziqian Jiang, Chuanli Wu, Xiuxun Han

**Affiliations:** 1 Institute of Optoelectronic Materials and Devices, School of Materials Science and Engineering, Jiangxi University of Science and Technology, Ganzhou 341000, China; 2 National Rare Earth Function Materials Innovation Center, Ganzhou 341100, China

**Keywords:** Cs_2_WCl_6_, Re^4+^ doping, NIR-II emission, broadband excitation feature

## Abstract

Halide double perovskites with near-infrared (NIR) emission are promising for optoelectronic applications. NIR-II (1000–1700 nm) emission, in particular, is attractive due to its strong tissue penetration, high spatial resolution, and low biological light damage risk. However, materials capable of NIR-II emission often require additional sensitizers and suffer from issues such as narrow emission bandwidth and low photoluminescence efficiency. In this work, we report a Re^4+^ doping strategy using Cs_2_WCl_6_, a vacancy–ordered double perovskite, to achieve efficient NIR-II emission. Spectroscopic and dynamic measurements reveal energy transfer between the Cs_2_WCl_6_ matrix and the Re^4+^ centers, resulting in efficient broadband NIR-II emission centered at 1345 nm (FWHM ≈ 87 nm), along with broad excitation ranging from 250 to 850 nm. The optimal NIR-II emission occurs at 1345 nm with a photoluminescence quantum yield (PLQY) of 29.83% when the Re^4+^ doping concentration is 1%. This work demonstrates an efficient, sensitizer-free method for achieving broadband NIR-II emission and provides a new material strategy for high–performance double perovskites NIR light sources.

## 1. Introduction

Halide double perovskites (A_2_B′B″X_6_) have emerged as promising candidates for solid–state lighting, photodetection, photovoltaics, and anticounterfeiting, owing to their structural diversity, compositional tunability, and outstanding stability [1,2,3]. In practice, their performance is frequently limited by B′/B″ site disorder and anti–site defects, which readily form deep nonradiative recombination centers [4,5]. To mitigate these issues, vacancy-–ordered double perovskites of the type A_2_BCl_6_ (A = Cs, Rb, K; B = Zr, Hf, Sn, W, Mo, etc.) have been developed as structurally robust alternatives [6,7,8,9,10,11]. In A_2_BCl_6_ compounds, intrinsic vacancy ordering simplifies the lattice, provides well-defined local coordination, and reduces the defect landscape [12,13]. Nevertheless, A_2_BCl_6_ luminescent perovskites remain constrained by moderate emission efficiency, weak photoluminescence, and, most critically, the lack of emission in the second near–infrared window (NIR-II, 1000–1700 nm). These limitations typically originate from indirect bandgap transitions or nominally direct transitions with partially forbidden character [14,15]. Therefore, effective design principles are urgently needed to activate and strengthen NIR-II emission in vacancy–ordered double perovskites.

Ion doping provides a versatile route to tailor and enhance the emission behavior of A_2_BCl_6_ hosts. Prior studies have achieved NIR-II photoluminescence by introducing lanthanide activators (e.g., Yb^3+^, Er^3+^, Nd^3+^) [16,17,18,19]. However, lanthanide–doped double perovskites generally show narrow–band NIR-II emission and often rely on additional sensitizers to promote energy transfer, while their quantum efficiencies remain modest. This inherent inefficiency mainly stems from forbidden (or partially allowed) 4f–4f transitions, which reduce radiative transition probabilities and ultimately limit brightness [20,21].

By contrast, Re^4+^, a transition–metal ion with a 5d^3^ electronic configuration, can produce near-infrared emission. In octahedral coordination, Re^4+^ shows characteristic NIR luminescence dictated by octahedral crystal–field splitting [22]. Notably, Cs_2_WCl_6_ exhibits intrinsic broadband emission over 800–1400 nm [23,24,25], which overlaps well with the 1000–1400 nm absorption band of Re^4+^. This spectral matching implies that incorporating Re^4+^ into Cs_2_WCl_6_ may allow the host’s UV/visible–excited emission to act as an intrinsic sensitizer, facilitating efficient W^4+^ → Re^4+^ energy transfer and thus enabling intense NIR-II emission (~1340 nm, assigned to the ^2^T_1g_ → ^4^A_2g_ transition). Moreover, because Re^4+^ is isovalent to W^4+^ in Cs_2_WCl_6_, it is expected to suppress defect formation and avoid trap states commonly associated with heterovalent doping, which is crucial for achieving high-efficiency perovskite emitters. Taken together, elucidating and harnessing W^4+^ → Re^4+^ energy transfer in Cs_2_WCl_6_ can provide fundamental insights and an effective pathway toward efficient broadband NIR-II luminescence.

Herein, Re^4+^-doped Cs_2_WCl_6_ single crystals were synthesized via a modified hydrothermal method, and their phase purity, morphology, and photophysical properties were systematically investigated. Upon 399 nm excitation, the crystals exhibited intense Re^4+^-derived broadband NIR-II emission centered at ~1345 nm with a full width at half–maximum (FWHM) of ~87 nm. Increasing the Re^4+^ content gradually quenched the host emission, evidencing highly efficient energy transfer from Cs_2_WCl_6_ to Re^4+^, with the NIR-II output maximized at 1% Re^4+^. Notably, Cs_2_WCl_6_:1% Re^4+^ achieved a near–infrared photoluminescence quantum yield (PLQY) of 29.83%. These results establish a vacancy–ordered double–perovskite host featuring intrinsic sensitization for efficient broadband NIR-II emission, opening opportunities in near–infrared lighting, optical communications, bioimaging, and nondestructive inspection.

## 2. Materials and Methods

Cesium chloride (CsCl, 99.9%) and tungsten (IV) chloride (WCl_4_, 85%) were purchased from Aladdin (Shanghai, China). Potassium hexachlororhenate (K_2_ReCl_6_, ≥99.9%) was purchased from Aladdin (Shanghai, China). Hydrochloric acid (HCl, 36~38%) and isopropanol (IPA, 99.7%) were of analytical grade and respectively purchased from Sinopharm Chemical Reagent Co. (Shanghai, China) and Damao Chemical Reagent Factory (Tianjin, China). All materials were used as received without further purification.

The pure Cs_2_WCl_6_ doped with x Re^4+^ (x = 0%, 0.1%, 0.5%, 0.8%, 1%, 2%, 3%) were prepared via hydrothermal reactions. The nominal doping concentration x in the perovskite represents the molar percentage (mol%) relative to the W site. Initially, accurately weighed amounts of CsCl (1 mmol) and WCl_4_ (0.5 mmol) and varying doping concentrations of K_2_ReCl_6_ (0.0005 mmol, 0.0025 mmol, 0.004 mmol, 0.005 mmol, 0.01 mmol, 0.015 mmol) were added to five 25 mL polytetrafluoroethylene (PTFE) liners. Subsequently, 10 mL of HCl was added to each liner, and the mixture solution was heated at 180 °C for 12 h in a stainless-steel Parr autoclave and then cooled to 30 °C at a rate of 15 °C/h. The precipitated products were then filtered out, washed with IPA, and dried in an oven at 60 °C.

## 3. Results and Discussion

**Figure 1:**
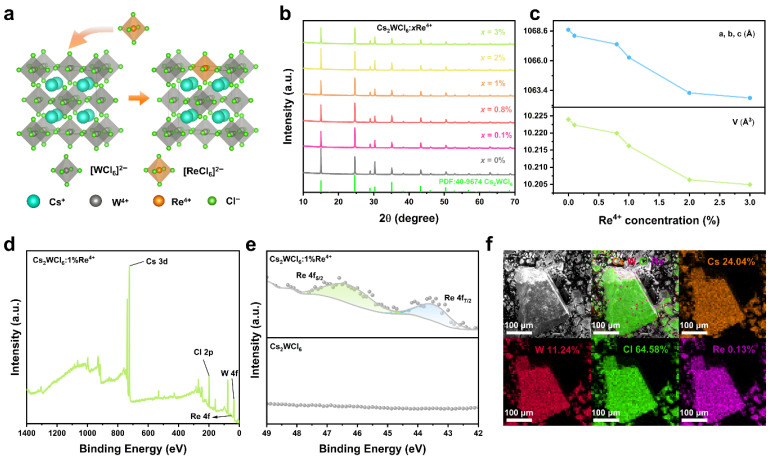
(**a**) Crystal structure of Cs_2_WCl_6_ and Re^4+^ doped Cs_2_WCl_6_. (**b**) XRD patterns of Cs_2_WCl_6_:xRe^4+^ (x = 0, 0.1%, 0.5%, 0.8%, 1%, 2%, and 3%). (**c**) Variation in the crystallographic parameters a, b, c, and V with x (Re^4+^) in Cs_2_WCl_6_. (**d**) Survey XPS spectra of Cs_2_WCl_6_:1%Re^4+^. (**e**) XPS spectra of Re element in Cs_2_WCl_6_ and Cs_2_WCl_6_:1%Re^4+^. (**f**) The EDS spectrum and the elemental mapping analysis of the Cs_2_WCl_6_:1%Re^4+^ powders.

Precise composition control was realized by systematically adjusting the nominal Re^4+^ fraction in the precursor mixture. As shown in Figure 1a, Cs_2_WCl_6_ features a vacancy–ordered framework composed of isolated [WCl_6_]^2−^ octahedra separated by interstitial Cs^+^ cations, and it can be regarded as a defect-derived variant of the three–dimensional perovskite lattice. Upon doping, Re^4+^ is expected to isovalently replace W^4+^ at the octahedral sites, entering the lattice as embedded [ReCl_6_]^2−^ units within the Cs_2_WCl_6_ framework.

Powder X-ray diffraction (XRD) patterns confirm that the hydrothermally prepared Cs_2_WCl_6_:xRe^4+^ samples are highly crystalline and show no detectable impurity phases (Figure 1b). All doped samples preserve the characteristic reflections of cubic Cs_2_WCl_6_ (space group $Fm\bar{3}m$), indicating a structurally robust host in which Re^4+^ incorporation does not induce an observable phase transition or lattice collapse [11]. A closer examination shows that the diffraction peaks shift systematically to higher 2θ as the Re^4+^ content increases, as highlighted by the evolution of the (220) reflection (Figure S1). This high–angle shift is attributed to lattice contraction caused by substitution of the slightly smaller Re^4+^ ion (r = 0.63 Å) for W^4+^ (r = 0.66 Å) [26]. This trend indicates that Re^4+^ preferentially occupies the W^4+^–based octahedral sites [27]. To further validate this assignment, Rietveld refinements were conducted for Cs_2_WCl_6_:xRe^4+^ (x = 0, 0.1%, 0.8%, 1%, 2%, and 3%) (Figure S2). The refinements results show low weighted–profile factors (*R*_wp_), profile factors (*R*_p_), and goodness–of–fit (*χ*^2^) values, which indicate excellent agreement between the experimental patterns and the structural model. The refined crystallographic parameters are summarized in Table S1. Consistently, the lattice constants and unit–cell volumes obtained from the refinements decrease monotonically with increasing Re^4+^ concentration (Figure 1c), further supporting substitutional incorporation of the smaller Re^4+^ ion at W^4+^ sites and the resulting lattice contraction.

Figure 1d shows the X–ray photoelectron spectroscopy (XPS) survey spectrum of Cs_2_WCl_6_:1%Re^4+^, where the characteristic signals of Cs, W, and Cl are readily observed. Because the dopant level is low (1%), Re–related features are not apparent in the survey spectrum; accordingly, a high-resolution scan was acquired over 42–49 eV (Figure 1e). Two clear Re 4f peaks (4f_7/2_ and 4f_5/2_) are observed at 43.6 and 46.5 eV, respectively, consistent with reported values and confirming that Re is predominantly in the +4 oxidation state [28]. Energy–dispersive spectroscopy (EDS) elemental mapping indicates a homogeneous distribution of all elements across the crystal (Figure 1f), giving atomic percentages (at%) of Cs, W, Cl, and Re of 24.04%, 11.24%, 64.58%, and 0.13%, respectively, which is overall consistent with the expected Cs_2_WCl_6_ stoichiometry. Quantitative ICP-OES analysis (Table S2) shows that the actual Re^4+^ incorporation closely matches the nominal feed ratios.

**Figure 2:**
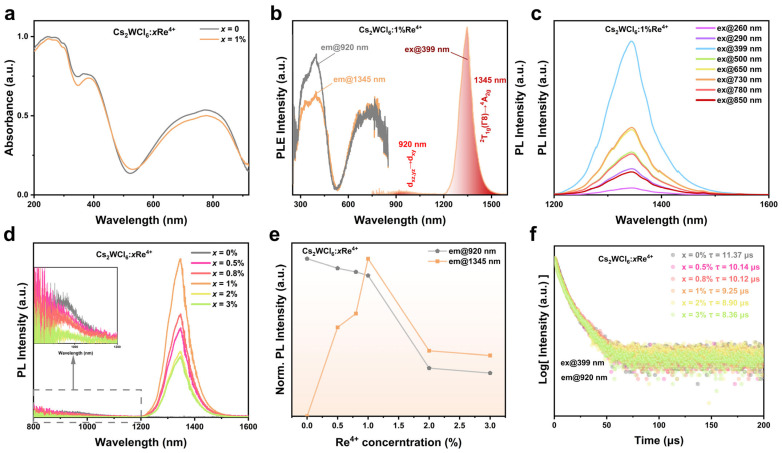
(**a**) UV-vis absorption spectra of Cs_2_WCl_6_ and Cs_2_WCl_6_:1%Re^4+^. (**b**) The excitation (**right**) and emission spectra (**left**) of Cs_2_WCl_6_:1%Re^4+^. (**c**) Excitation wavelength-dependent photoluminescence of Cs_2_WCl_6_:1%Re^4+^ at 1345 nm (^2^T_1g_ → ^4^A_2g_). (**d**) Emission spectra, (**e**) normalized PL intensity curves and (**f**) normalized decay curves monitored at 920 nm for Cs_2_WCl_6_:xRe^4+^ (x = 0, 0.5%, 0.8%, 1%, 2%, and 3%) under 399 nm excitation at room temperature.

Through the ultraviolet–visible (UV-Vis) absorption spectra and steady–state fluorescence representation, systematic investigations were conducted on the photophysical properties of Cs_2_WCl_6_:xRe^4+^, and the potential luminescence mechanisms were explored. Firstly, the optical absorption behavior of Cs_2_WCl_6_ doped with Re^4+^ was examined at room temperature. As shown in Figure 2a, compared with the Cs_2_WCl_6_ host, the doped sample exhibits essentially identical absorption features from 200 to 900 nm, while characteristic Re^4+^ absorption appears in the 1000–1400 nm region (Figure S3). Referencing previous reports [10,22,24,29], the spectral features can be assigned as follows: absorption below ~500 nm mainly arises from parity–allowed ligand–to–metal charge–transfer (LMCT) transitions; the sub-band absorption from 500 to 900 nm corresponds to the dominant excitation channels responsible for the host near-infrared emission; and the bands between 1000 and 1400 nm are attributed to Re^4+^ crystal–field–related absorptions involving the Γ_6_(^2^T_1g_), Γ_8_(^2^E_g_), and Γ_8_(^2^T_1g_) manifolds in an octahedral coordination environment.

To probe the coupling between the host and Re^4+^ and clarify possible luminescence pathways, excitation and emission spectra of Cs_2_WCl_6_:1%Re^4+^ were collected (Figure 2b). Under 399 nm excitation, the PL spectrum displays two broad near–infrared bands spanning 800–1600 nm, centered at 920 nm (NIR-I, FWHM ≈ 156 nm) and 1345 nm (NIR-II, FWHM ≈ 87 nm). The mainstream literature attributes the host emission at 920 nm and the Re^4+^ emission at 1345 nm to d–d transitions within the octahedral coordination crystal fields of [WCl_6_]^2−^ and [ReCl_6_]^2−^, specifically the d_xz,yz_ → d_xy_ transition of W^4+^ and the ^2^T_1g_(Γ_8_) → ^4^A_2g_ transition of Re^4+^ [30,31]. Notably, excitation spectra monitored at 920 and 1345 nm are nearly identical, showing a broad excitation envelope from 250 to 850 nm that closely matches the absorption profile. Emission spectra acquired at different excitation wavelengths (Figure 2c and Figure S4) show intensity variations consistent with the excitation spectra. Collectively, these results suggest that Re^4+^ luminescence does not arise from an independent, purely localized excitation process but is instead strongly coupled to host absorption/emission dynamics, consistent with a resonant energy-transfer pathway between the two species [32].

This interpretation is further supported by the room–temperature dependence of PL intensity on Re^4+^ concentration (Figure 2d). As the Re^4+^ content increases, the NIR-I band at 920 nm is gradually quenched, whereas the NIR-II band at 1345 nm increases initially and then decreases, peaking at 1% doping (Figure 2e). At higher dopant levels, enhanced Re^4+^-Re^4+^ energy migration induces concentration quenching, and excitation energy is more readily transferred to nonradiative sinks (e.g., lattice distortions and surface–related trap states), resulting in reduced emission [31,33]. In addition, the PL stability of the Cs_2_WCl_6_:1%Re^4+^ crystal was evaluated after storage in air for 7 days at a relative humidity of (30 ± 10) % and room temperature of (20 ± 5) °C. The results show that the PL intensity decreased by only ~8% compared with the freshly prepared crystal (Figure S5), indicating that the material possesses good environmental stability.

To understand how Re^4+^ incorporation affects luminescence kinetics, time–resolved photoluminescence (TRPL) measurements were carried out on Cs_2_WCl_6_:xRe^4+^ (x = 0, 0.1%, 0.5%, 0.8%, 1%, 2%, and 3%). The decay traces, which were monitored at 920 nm under 399 nm pulsed xenon–lamp excitation, are presented in Figure 2f. The 920 nm lifetime decreases monotonically as the Re^4+^ content increases, providing additional evidence for resonant host–to–Re^4+^ energy transfer that can be described by a Förster–Dexter framework. Under the same excitation conditions, the decay curve of the 1345 nm emission from Cs_2_WCl_6_:1%Re^4+^ is shown in Figure S6. The extracted lifetime is comparable to those reported for other Re^4+^–doped systems [22,28,31], consistent with intrinsic Re^4+^ emission on the millisecond timescale.

**Figure 3:**
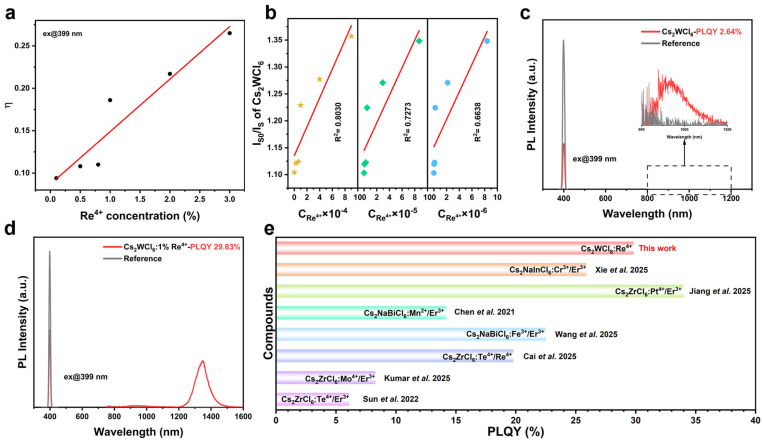
(**a**) Dependence of energy efficiency *η* on Re^4+^ doping concentration, and (**b**) dependence of *I*_S0_/*I*_S_ of Cs_2_WCl_6_ on C_Re_^4+^ × 10^2^, C_Re_^4+^ × 10^8/3^, C_Re_^4+^ × 10^10/3^. (**c**,**d**) PLQY of Cs_2_WCl_6_ and Cs_2_WCl_6_:1%Re^4+^. (**e**) A bar chart plotted based on the PLQY values reported in previously published literature [15,31,37,38,39,40,41] on double perovskite materials.

The energy–transfer efficiency from the host to Re^4+^ is given by the following equation [34]:

(1) $$\eta =1-\frac{\tau }{\tau_{0}}\times 100\%$$

where *τ* and *τ*_0_ are the host (donor) photoluminescence lifetimes in the absence and presence of Re^4+^ (acceptor), respectively. As shown in Figure 3a, *η* increases strongly with Re^4+^ content and reaches a maximum value of 26.5% at 3% Re^4+^. The efficient host → Re^4+^ energy transfer can be rationalized by the substitution of Re^4+^ for W^4+^ in the Cs_2_WCl_6_, which shortens the donor–acceptor distance and increases the transfer probability. However, the energy transfer efficiency monotonously ascends with a continuous increase in Re^4+^ concentration while the increasing rate decreases. According to Förster–Dexter’s energy transfer expression of multipolar interaction, the energy transfer mechanism from Cs_2_WCl_6_ to Re^4+^ ions in this host should occur via electric multipole–multipole interactions.

To identify the dominant interaction, we analyzed the energy transfer mechanism using the Förster–Dexter multipolar interaction model. In this model, the dependence of donor emission quenching on dopant concentration can be described by the following formula [35,36]:

(2) $$\frac{I_{S0}}{I_{S}}\propto C^{\alpha /3}$$

where *C* is the Re^4+^ concentration, *I*_S0_ is the luminescence intensity in the absence of Re^4+^, and *I*_S_ is the luminescence intensity in the presence of Re^4+^. *α* = 6, 8, and 10 correspond to dipole–dipole, dipole–quadrupole, and quadrupole–quadrupole interactions, respectively. As shown in Figure 3b, least-squares fits of *I*_S0_/*I*_S_ versus *C^α^*^/3^ (*α* = 6, 8, and 10) show the best linearity for *α* = 6, giving the highest residual (R^2^ = 0.803). These results indicate that host–to–Re^4+^ energy transfer is dominated by dipole–dipole coupling.

We then quantified the near-infrared photoluminescence quantum yields (PLQYs) of Cs_2_WCl_6_ and Cs_2_WCl_6_:1% Re^4+^ under 399 nm excitation (Figure 3c,d). The undoped host exhibits predominantly NIR-I emission with a low PLQY of 2.64%. By contrast, the optimally doped sample retains only a weak residual NIR-I band while exhibiting markedly enhanced NIR-II emission, achieving a total PLQY of 29.83%. Given the emission composition, the total PLQY can be taken as a close approximation of the NIR-II quantum efficiency. Notably, relative to previously reported lead-free perovskite systems emitting in the NIR-II window, the NIR-II PLQY achieved here is among the higher values (Figure 3e and Table S3) [15,31,37,38,39,40,41].

**Figure 4:**
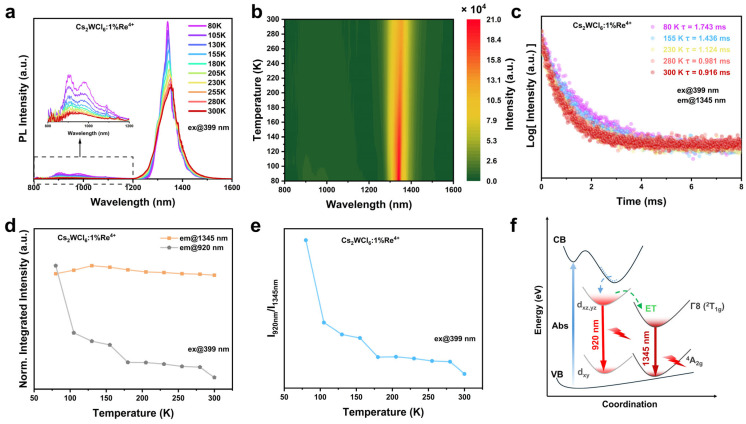
(**a**) Temperature-dependent PL emission properties of Cs_2_WCl_6_:1%Re^4+^. (**b**) 2D pseudo-color contour temperature-dependent PL plots of Cs_2_WCl_6_:1%Re^4+^. (**c**) Decay traces of Cs_2_WCl_6_:1%Re^4+^ at different temperature for emission corresponding to Re^4+^ d-d transition at 1345 nm (^2^T_1g_ → ^4^A_2g_). (**d**) Normalized integrated intensities of the W^4+^ and Re^4+^ emission at different temperatures. (**e**) Variation in integrated emission intensity ratio of W^4+^ (920 nm) and Re^4+^ (1345 nm) of Cs_2_WCl_6_:1% Re^4+^ with temperature. (**f**) Schematic energy diagram of the proposed ET model in Re^4+^ doped Cs_2_WCl_6_.

We systematically investigated the temperature-dependent evolution of the emission spectra and lifetime decay curves. Temperature–dependent photoluminescence (PL) spectra of Cs_2_WCl_6_:1% Re^4+^ collected from 80 to 300 K are shown in Figure 4a. As temperature increases, the PL intensity decreases monotonically, which is readily visualized in temperature–dependent pseudo–color maps (Figure 4b and Figure S7). This behavior is characteristic of thermal quenching, where elevated temperatures activate nonradiative recombination pathways [10,31]. At low temperature, the W^4+^–related host emission shows an apparent splitting into three asymmetric components (inset of Figure 4a), attributable to vibronic coupling of the d–d transition [41]. Upon heating, these features broaden and gradually coalesce into a single band. A similar temperature–driven spectral evolution is observed for undoped Cs_2_WCl_6_ over 80–300 K (Figure S8). The Re^4+^ emission exhibits a similar trend: at low temperature, reduced phonon participation partially resolves fine/vibronic structure, whereas at higher temperature increased phonon scattering enhances homogeneous broadening and modifies the relative contributions of vibronic sidebands, yielding a smoother and broader band. Notably, the Re^4+^ emission maximum shows a clear redshift upon warming, indicating an increasing contribution from thermally activated vibronic relaxation. This temperature–dependent redshift and line–shape evolution can be rationalized in terms of strengthened vibronic coupling and possible dynamic Jahn–Teller (dJT) effects [42], which may occur when the relevant crystal–field manifolds retain (quasi-)orbital degeneracy. With increasing temperature, the growing phonon population enhances thermal activation of vibrational states associated with local distortions and strengthens electron–phonon interactions, thereby redistributing spectral weight toward lower–energy vibronic components and ultimately shifting the emission maximum to lower energy. Temperature–dependent line-shape evolution of this type has been widely discussed for halide crystals doped with [ReCl_6_]^2−^ [28,43,44,45].

The corresponding temperature dependence of the emission lifetimes for Cs_2_WCl_6_:1% Re^4+^ is presented in Figure 4c and Figure S9a. The lifetime of the Re^4+^ ^2^T_1g_(Γ_8_) excited manifold decreases monotonically with increasing temperature, consistent with enhanced nonradiative relaxation driven by stronger vibronic coupling within the NIR-II emission band. The temperature–dependent evolution of the W^4+^ lifetime is consistent with that of the PL intensity, both showing a monotonic decrease with increasing temperature. The detailed lifetime variation is presented in Figure S9b, further highlighting the increasing contribution of thermally activated nonradiative pathways to luminescence decay.

To more fully delineate the sensitization pathway in Re^4+^–doped Cs_2_WCl_6_, we compare the normalized integrated PL intensities at 920 and 1345 nm (Figure 4d). With increasing temperature, the W^4+^–related emission decreases markedly, which may arise from both enhanced thermally enhanced W^4+^ → Re^4+^ energy transfer (ET) and enhanced nonradiative depopulation. In contrast, the Re^4+^ emission increases monotonically from 80 to 130 K and then gradually decreases, providing initial evidence for a thermally enhanced ET component. Consistently, Figure 4e shows the temperature dependence of the integrated intensity ratio I_920_/I_1345_ (W^4+^ vs. Re^4+^ emission). The pronounced drop in this ratio from 80 to 130 K indicates that warming preferentially promotes Re^4+^ emission while W^4+^ emission, further supporting thermally enhanced ET. Such behavior is commonly observed in sensitized luminescent systems [31,41]. Collectively, these results support a mechanism in which Re^4+^ emission is predominantly enabled through host sensitization.

Figure 4f shows the schematic mechanism of energy transfer between the host and Re^4+^ ions, which was drawn based on the experimental results mentioned above and relevant literature. Upon 399 nm excitation, electrons are promoted from the valence band to the conduction band of the Cs_2_WCl_6_ host. Subsequently, carriers undergo non-radiative relaxation and structural reorganization to populate the W^4+^ excited state, leading to radiative emission centered at 920 nm via the d_xz,yz_ → d_xy_ transitions. Concurrently, energy from the W^4+^ excited state can be transferred not only to the d_xy_ state via relaxation but also to the ^2^T_1g_ level of Re^4+^. Subsequently, this energy relaxes from ^2^T_1g_ to the ground state ^4^A_2g_, producing NIR-II emission centered at 1345 nm through the ^2^T_1g_ → ^4^A_2g_ radiative transition, ultimately achieving highly efficient NIR-II luminescence.

## 4. Conclusions

This study successfully synthesized a series of Cs_2_WCl_6_:xRe^4+^ (x = 0–3%) perovskite single crystals via the hydrothermal method, and their crystal structures and near-infrared luminescence properties were systematically investigated. Owing to the broad excitation band of the Cs_2_WCl_6_ host (250–850 nm), the doped samples display two broadband near-infrared emissions under various excitation conditions: an NIR-I band at 920 nm arising from the d_xz,yz_ → d_xy_ transition (FWHM ≈ 156 nm) and an NIR-II band near 1345 nm attributed to the Re^4+^ ^2^T_1g_ → ^4^A_2g_ transition (FWHM ≈ 87 nm). Enabled by efficient host → Re^4+^ energy transfer, the NIR-II emission is markedly enhanced, reaching a maximum at 1% Re^4+^ with a near-infrared photoluminescence quantum yield (PLQY) of 29.83%. Moreover, lifetime analysis, multipolar interaction fitting, and temperature-dependent measurements indicate that Re^4+^ NIR-II emission is predominantly enabled via host sensitization. In summary, Cs_2_WCl_6_ acts as an efficient sensitizing host that enables broadband NIR-II emission with a wide excitation bandwidth via transition–metal–ion doping. This work establishes a new material platform and mechanistic basis for expanding the application of vacancy-ordered/double-perovskite emitters in near-infrared illumination, optical communication, bioimaging, and nondestructive testing.

## Data Availability

The original contributions presented in this study are included in the article/Supplementary Materials. Further inquiries can be directed to the corresponding authors.

## Supplementary Materials

The following supporting information can be downloaded at https://www.mdpi.com/article/10.3390/nano16070400/s1. Table S1: Rietveld refinement result of Cs_2_WCl_6_:xRe^4+^ samples (x = 0, 0.1%, 0.8%, 1%, 2%, 3%); Table S2: Schemes of different molar ratios of Cs_2_WCl_6_ to Re^4+^ for the synthesis of Cs_2_WCl_6_:Re^4+^ and the actual doping amount of Re^4+^ measured by the ICP-OES; Table S3: Comparison of the PLQY values of Cs_2_WCl_6_:1%Re^4+^ double perovskite with previously developed NIR-emitting halide double perovskites; Figure S1: Enlarged PXRD patterns of the (220) reflection of Cs_2_WCl_6_:xRe^4+^ (x = 0, 0.1%, 0.8%, 1%, 2%, 3%); Figure S2: XRD Rietveld refinement of Cs_2_WCl_6_:xRe^4+^ (x = 0, 0.1%, 0.8%, 1%, 2%, 3%); Figure S3: Absorbance spectra of Cs_2_WCl_6_:xRe^4+^ (x = 0, 1%); Figure S4: PL spectra of Cs_2_WCl_6_:1%Re^4+^ under excitation at 310 nm and 399 nm, respectively; Figure S5: PL spectra of the Cs_2_WCl_6_:1%Re^4+^ sample before and after storage in air for 7 days min at a relative humidity of (30 ± 10)% and room temperature of (20 ± 5) °C; Figure S6: Decay curves of Re^4+^ in Cs_2_WCl_6_:1%Re^4+^; Figure S7: 2D pseudo-color contour temperature-dependent PL plots of Cs_2_WCl_6_:1%Re^4+^ excited at 399 nm; Figure S8: Temperature-dependent emission spectra of Cs_2_WCl_6_ excited at 399 nm; Figure S9: (a) Decay curves and (b) variation in Cs_2_WCl_6_:1%Re^4+^ at the 80–300 K. K. References [15,31,37,38,39,40,41] are cited in the supplementary materials.
