# Supplementary material for: Broadband NIR-II Emission with Wide Excitation Range in Cs2WCl6 Double Perovskites Utilizing Re4+ Doping
Source: Nanomaterials (Basel). 2026 Mar 26;16(7):400. doi: 10.3390/nano16070400 (PMC13074438; doi:10.3390/nano16070400)
Supplement: Supplementary file 1 [file nanomaterials-16-00400-s001.zip › nanomaterials-4172587-supplementary.pdf]

nanomaterials

MDPI

Supporting Information

Broadband NIR-II Emission with Wide Excitation Range in Cs_2WCl_6 Double Perovskites Utilizing Re^{4+} Doping

Yu Xiao [1,2], Xiaobo Hu [1,2], Ziqian Jiang [1], Chuanli Wu [1,2,*] and Xiuxun Han [1,2,*]

[1] Institute of Optoelectronic Materials and Devices, School of Materials Science and Engineering, Jiangxi University of Science and Technology, Ganzhou 341000, China
[2] National Rare Earth Function Materials Innovation Center, Ganzhou 341100, China
* Correspondence: chuanli_wu@hotmail.com (C.W.); xxhan@jxust.edu.cn (X.H.)

Measurement and Characterization

The crystal structures were observed using X-ray diffractometer (XRD, TD-3700) with Cu Kα1 radiation (λ = 0.154187 nm) in a scan range of 10-90°. Rietveld refinement was performed using the GSAS2 software package. The morphological images of the samples were recorded by SEM (Phenom Pure, Netherlands), and energy-dispersive spectrometry (EDS) spectroscopy on a Phenom Pure scanning electron microscope operated at 20 kV. The absorption spectra were translated from UV-vis diffuse reflectance spectra, which were recorded using UV-2600 spectrometer (Shimadzu, Japan). Photoluminescence (PL) excitation, emission spectra, PL decay curves and photoluminescence quantum yields (PLQYs) were measured on the FLS980 spectrometer (Edinburgh, UK). The temperature-dependent PL spectra was tested by the FLS1000 spectrophotometer (Edinburgh, UK) equipped with NIR photomultiplier. The X-ray photoelectron spectroscopy (XPS) spectrum was collected using 5000 VersaProbe III (PHI, Japan). The elemental composition of samples was studied using 5110 ICP-OES (Agilent technologies, USA).

 https://doi.org/10.3390/nano16070400

Table S1. Rietveld refinement result of Cs_2WCl_6:xRe^{4+} samples (x = 0, 0.1%, 0.8%, 1%, 2%, 3%).

x/ mol%	Space group	a, b, c (Å)	V (Å^3)	R_p (%)	R_{wp} (%)	X^2
0	$Fm\bar{3}m$	10.22397	1068.707	7.13	9.63	1.20
0.1	$Fm\bar{3}m$	10.22233	1068.193	7.40	9.71	1.17
0.8	$Fm\bar{3}m$	10.21993	1067.441	6.70	8.84	1.08
1	$Fm\bar{3}m$	10.21623	1066.282	7.39	9.70	1.16
2	$Fm\bar{3}m$	10.20635	1063.192	7.99	11.57	1.42
3	$Fm\bar{3}m$	10.20493	1062.746	7.89	10.45	1.25

Table S2. Schemes of different molar ratios of Cs_2WCl_6 to Re^{4+} for the synthesis of Cs_2WCl_6:Re^{4+} and the actual doping amount of Re^{4+} measured by the ICP-OES.

Sample	W^{4+} : Re^{4+} (Molar ratio)	Actual ratio of Re^{4+} (mol%)
1	100:0.8	0.77
2	100:1	1.27
3	100:2	1.34

https://doi.org/10.3390/nano16070400

Table S3. Comparison of the PLQY values of Cs_2WCl_6:1%Re^{4+} double perovskite with previously developed NIR-emitting halide double perovskites.

Compounds	Excitation (nm)	Emission (nm)	PLQY	Ref
Cs_2ZrCl_6:Te^{4+}/Er^{3+}	392	1539	6.1%	[1]
$Cs_2NaBiCl_6$: Mn^{2+}/Er^{3+}	360	1540	14.2%	[2]
Cs_2ZrCl_6:Te^{4+}/Re^{4+}	290	1340	19.8%	[3]
$Cs_2NaInCl_6$: Cr^{3+}/Er^{3+}	290	1540	25.88%	[4]
Cs_2ZrCl_6:Pt^{4+}/Er^{3+}	254	1540	34%	[5]
$Cs_2NaBiCl_6$: Fe^{3+}/Er^{3+}	450	1541	22.5%	[6]
Cs_2ZrCl_6:Mo^{4+}/Er^{3+}	360	1543	8.3%	[7]
Cs_2WCl_6:Re^{4+}	399	1345	29.83%	This work

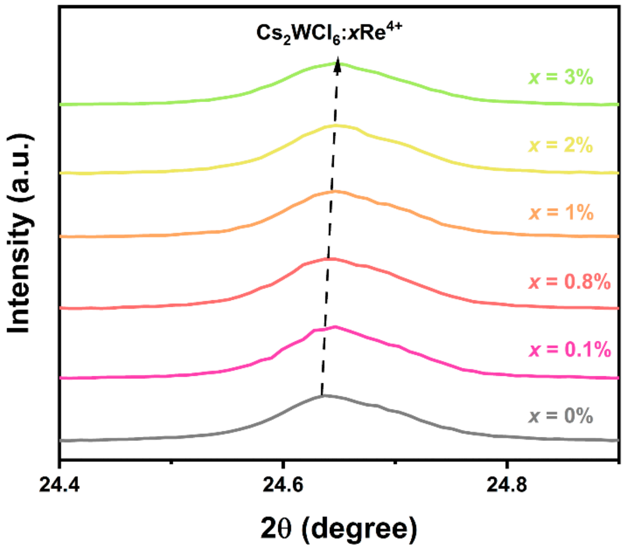

Figure S1. Enlarged PXRD patterns of the (220) reflection of Cs_2WCl_6:xRe^{4+} (x = 0, 0.1%, 0.8%, 1%, 2%, 3%).

https://doi.org/10.3390/nano16070400

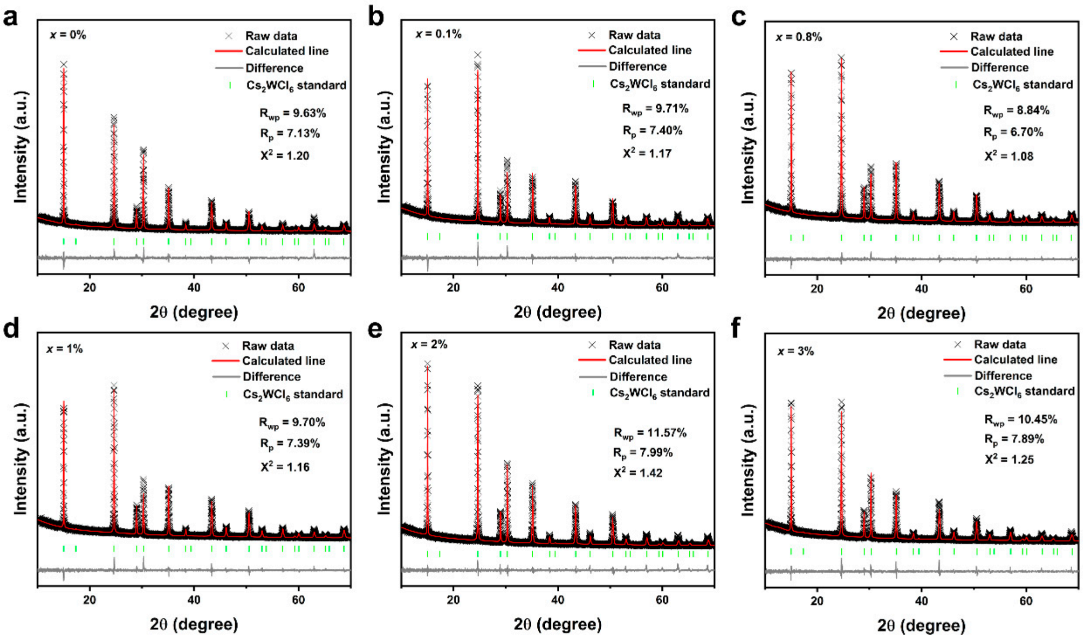

Figure S2. XRD Rietveld refinement of Cs_2WCl_6:xRe^{4+} (x = 0, 0.1%, 0.8%, 1%, 2%, 3%).

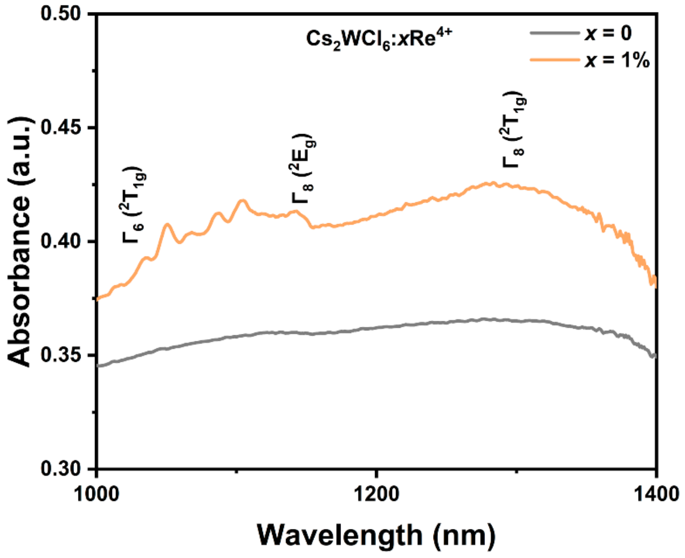

Figure S3. Absorbance spectra of Cs_2WCl_6:xRe^{4+} (x = 0, 1%).

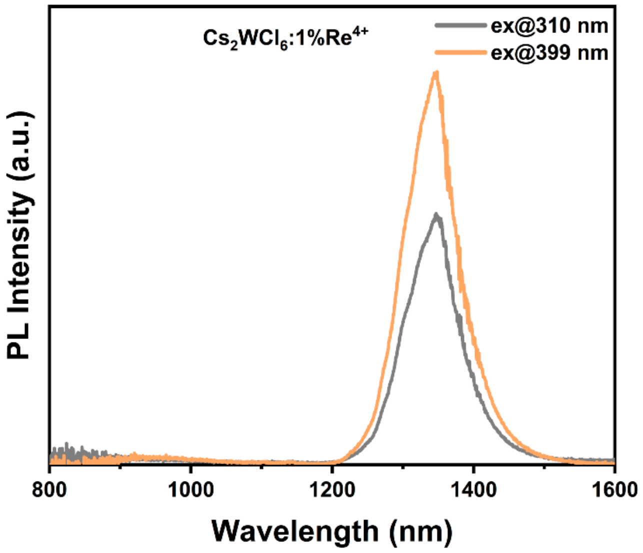

Figure S4. PL spectra of Cs_2WCl_6:1%Re^{4+} under excitation at 310 nm and 399 nm, respectively.

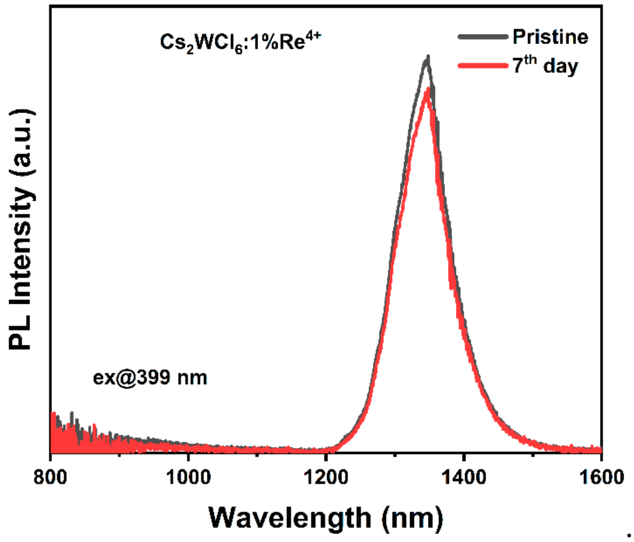

Figure S5. PL spectra of the Cs_2WCl_6:1%Re^{4+} sample before and after storage in air for 7 days at a relative humidity of (30 ± 10)% and room temperature of (20 ± 5) °C.

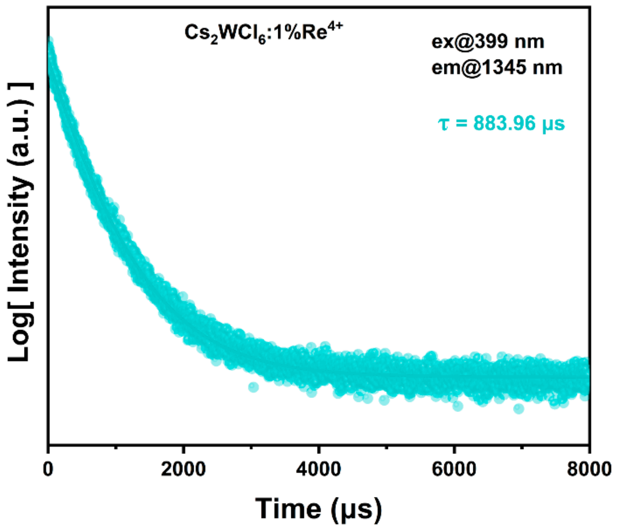

Figure S6. Decay curves of Re^{4+} in Cs_2WCl_6:1%Re^{4+}.

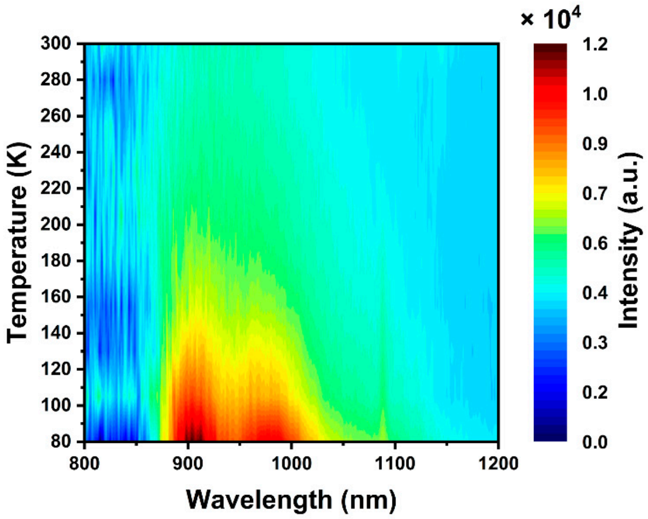

Figure S7. 2D pseudo-color contour temperature-dependent PL plots of Cs_2WCl_6:1%Re^{4+} excited at 399 nm.

https://doi.org/10.3390/nano16070400

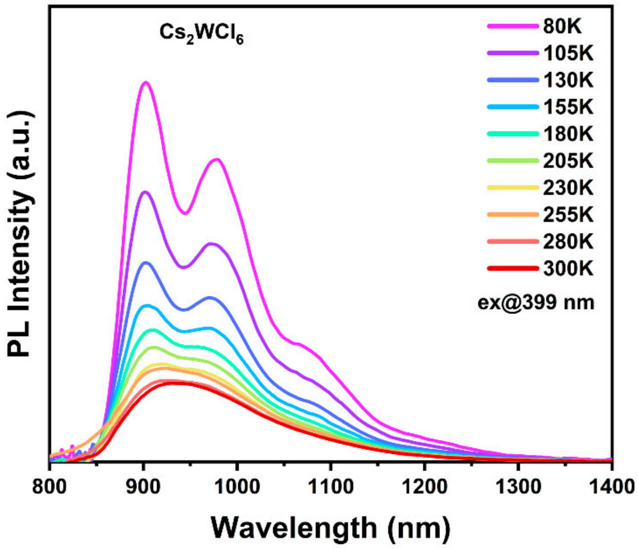

Figure S8. Temperature-dependent emission spectra of Cs_2WCl_6 excited at 399 nm.

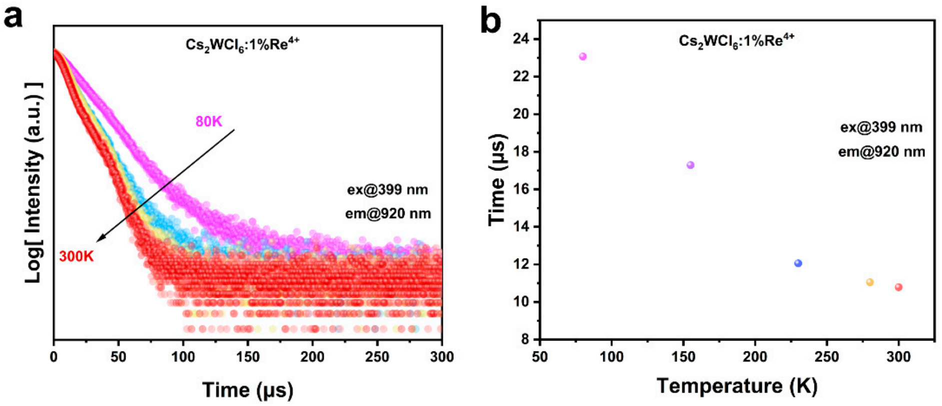

Figure S9. (a) Normalized decay curves and **(b)** variation of Cs_2WCl_6:1%Re^{4+} at the 80–300 K.

Reference

1. Sun, J.; Zheng, W.; Huang, P.; Zhang, M.; Zhang, W.; Deng, Z.; Yu, S.; Jin, M.; Chen, X. Efficient near-infrared luminescence in lanthanide-doped vacancy-ordered double perovskite Cs_2ZrCl_6 phosphors via Te^{4+} sensitization. *Angew. Chem.* **2022**, *134*, e202201993.
2. Chen, B.; Guo, Y.; Wang, Y.; Liu, Z.; Wei, Q.; Wang, S.; Rogach, A.L.; Xing, G.; Shi, P.; Wang, F. Multiexcitonic emission in zero-dimensional Cs_2ZrCl_6:Sb^{3+} perovskite crystals. *J. Am. Chem. Soc.* **2021**, *143*, 17599–17606.
3. Cai, P.; Li, B.; Zhan, Y.; Feng, X.; Pu, X.; Wang, L.; Chen, Z.; Liu, Z.; Bi, S. Re^{4+}/Te^{4+} co-doped Cs_2ZrCl_6 double perovskite micro-crystals: Broadening excitation range and boosting luminescent performance for near-infrared lighting and non-destructive quality inspection. *Mater. Today Chem.* **2025**, *48*, 102954.
4. Xie, H.; Fu, H.; Du, Z.; Tong, L.; Jiang, J.; Jiang, X.; Zhao, J.; Yang, W.; Zheng, J. Cr^{3+}-induced broadband near-infrared I combined with near-infrared II emission via rare earth co-doping in $Cs_2NaInCl_6$ for multifunctional detection. *J. Mater. Chem. C* **2025**, *13*, 14648–14656.
5. Jiang, C.; Liu, Q.; Li, L.; Li, K.; Feng, Y.; Fu, Y.; Li, Y.; Qian, X.; Wei, B.; Du, P. Tailoring of visible-NIR-II luminescence in Pt^{4+}/Er^{3+}-codoped Cs_2ZrCl_6 double perovskite phosphors via energy transfer engineering for diversified applications. *Laser Photonics Rev.* **2025**, *19*, 2401940.

https://doi.org/10.3390/nano16070400

6. Wang, Y.; Lou, B.; Dang, P.; Zhang, G.; Wan, Y.; Tian, L.; Lian, H.; Hou, Z.; Ma, C.; Li, G. Enhancement of NIR-II emission of Er^{3+} by doping Fe^{3+} in double perovskites: Multimode luminescence for versatile optoelectronic applications. *Angew. Chem.* **2025**, *137*, e202416021.
7. Kumar, S.; Lamba, R.S.; Singh, R.; Jha, V.; Yadav, J.; Sapra, S. Efficient near-infrared luminescence with broad-band sensitization in Mo^{4+}–Er^{3+} co-doped Cs_2ZrCl_6 vacancy-ordered double perovskites. *Small* **2025**, *21*, e07964.

.
